# Using High-Resolution Vessel Wall Magnetic Resonance Images in a Patient of Intracranial Artery Dissection Related Acute Infarction

**DOI:** 10.3390/diagnostics14141463

**Published:** 2024-07-09

**Authors:** Chia-Yu Lin, Hung-Chieh Chen, Yu-Hsuan Wu

**Affiliations:** 1 Division of Neuroradiology, Department of Radiology, Taichung Veterans General Hospital, Taichung 40705, Taiwan; 2 School of Medicine, National Yang-Ming Chiao Tung University, Taipei 11221, Taiwan; 3 Division of Neurology, Neurological Institute, Taichung Veterans General Hospital, Taichung 40705, Taiwan

**Keywords:** vessel wall image, magnetic resonance imaging (MRI), dissection, intra-arterial thrombectomy (IA thrombectomy)

## Abstract

Acute ischemic stroke in young adults typically carries significant implications for morbidity, mortality, and long-term disability. In this study, we describe the case of a 34-year-old male with no prior medical history who presented with symptoms of right-sided weakness and slurred speech, suggesting an acute ischemic stroke. Initial CT angiography revealed an occlusion in the left M2 segment middle cerebral artery (MCA). The occlusion was successfully recanalized through emergent endovascular thrombectomy, which also identified a dissection as the cause of the stroke. Follow-up assessments at 3 days and three months, which included advanced vessel wall MRI, highlighted the critical role of intracranial artery dissection in strokes among young adults and provided essential images for ongoing evaluation.

Acute ischemic stroke in young adults, typically defined as individuals between the ages of 18 and 50 years, represents a medical condition that, while less common than in older populations, carries significant implications for morbidity, mortality, and long-term disability [1]. Strokes in young individuals often have similar risk factors to those in older adults: high blood pressure, diabetes, high cholesterol, and obesity. However, young adults with acute ischemic stroke may have more unique and sometimes genetic conditions such as arterial dissections [2]; coagulopathies [3]; Moyamoya disease [4]; genetic syndromes like Marfan syndrome; embolisms from heart anomalies; infections; or they may be taking certain medications [5]. The impact of acute stroke in this demographic is profound, not only due to the immediate health concerns but also because of the long-term consequences for the individual’s quality of life, employment, and social contributions [1]. Diagnosing and managing acute ischemic stroke in young adults requires a tailored approach that considers the wide spectrum of potential causes and the need for comprehensive rehabilitation services to address the physical, cognitive, and emotional challenges that may arise.

Intracranial artery dissection is identified as a notable cause of stroke, especially among young and middle-aged populations [2]. The critical role of advanced diagnostic imaging techniques, including high-resolution magnetic resonance imaging (MRI) and digital subtraction angiography, is emphasized in the accurate detection of intracranial arterial dissection.

A 34-year-old male right-handed police officer, with no significant medical history, presented himself to our emergency room (ER) with acute onset symptoms of unsteady gait, slurred speech, and right-sided weakness. Initial examination in the ER revealed partial Broca’s aphasia, right central facial palsy, and a National Institutes of Health Stroke Scale (NIHSS) score of 9. Emergent CT angiography revealed an occlusion in the left-middle cerebral artery (MCA). After discussing the situation with his family, he underwent endovascular thrombectomy (EVT) (Figure 1). During the EVT, dissection of the left MCA with acute vessel occlusion was diagnosed as the etiology of this patient (Figure 2 and Figure 3).

A follow-up brain MRI performed 3 days after EVT confirmed acute infarction in the left frontoparietal region and left insular cortex, along with a small area of hemorrhagic transformation. Interestingly, vessel wall MRI revealed a distinct enhancement pattern. A faint high signal on pre-contrast T1-weighted imaging (T1WI) along the left proximal M2 segment of the MCA vessel wall was observed due to arterial dissection, with eccentric enhancement in the corresponding region following gadolinium injection. (Figure 4A,B) Additionally, concentric vessel wall enhancement in the distal left MCA vessel wall, distal to the stent retriever location, was noted without abnormal signal on pre-contrast T1WI, likely due to a post-EVT induced inflammatory process. (Figure 5A,B) At the three-month follow-up MRI, the previously observed faint high signal on T1WI in the left proximal M2 segment of the MCA had almost disappeared, indicating that the dissection healed over time. (Figure 4C,D) Additionally, no contrast enhancement of the vessel wall was detected in the follow-up MRI. (Figure 5C,D).

Internal carotid artery dissection is a crucial cause of stroke in the young, arising from a tear allowing blood into the arterial wall. Except presenting as occlusion of the affected artery, some patients presented with subarachnoid hemorrhage [7]. Diagnosis of this condition is challenging due to varied symptoms, and treatment typically involves antiplatelet therapy, with endovascular interventions for resistant cases.

For our patients, the cardiac evaluations and a young stroke survey showed that the autoimmune antibodies, protein C and protein S levels, and homocysteine in this patient were within normal limits. The patient had normal blood pressure. The patient showed significant improvement in right-sided weakness after EVT, aspirin treatment, and a further rehabilitation program.

Vessel wall MRI (VW-MRI) has emerged as a pivotal imaging modality for diagnosing intracranial arterial diseases, offering unparalleled insights into the arterial wall’s pathology [4]. Various sequences should be scanned using either multiplanar 2D or volumetric 3D captures in VW-MRI to obtain high spatial resolution images and to effectively eliminate signals from luminal blood and CSF. We routinely used a 3 Tesla MR machine containing 3D pre- and post-Gd black blood T1WI images and 3D T2WI images [8]. This technique allows for the direct visualization of critical features for dissection, such as the intimal flap, double lumen, intramural hematoma, and abnormal arterial wall thickening and enhancement on pre-contrast T1WI [6]. The intimal flap, indicative of the inner arterial layer’s separation, and the double lumen phenomenon, resulting from blood infiltration into the arterial wall, are hallmark signs of dissection. Intramural hematoma, visible as high signal intensity on T1-weighted VW-MRI sequences, arises from bleeding beneath the vessel’s inner lining. However, a high T1 signal in the wall should be evaluated carefully and neuroradiologist should look at both T1 and T2 simultaneously to prevent misdiagnosis of turbulent flow [9]. Additionally, post-Gd T1WI images should also be reviewed, as they often reveal abnormal thickening and enhancement associated with inflammation within the artery wall [10,11]. Over time, VW-MRI can capture the dynamic changes in the dissected artery, including the resolution of the hematoma, changes in the vessel wall’s appearance, and the potential recanalization of the artery. These temporal changes are crucial for monitoring disease progression and guiding therapeutic interventions.

The observed concentric enhancement in the distal left MCA, beyond the location of the stent retriever, aligns with findings reported in previous studies [12]. These studies have noted that patients undergoing intra-arterial treatment often show a higher incidence of ipsilateral concentric contrast enhancement, as opposed to the contralateral side. Such a pattern may result from the direct mechanical impact exerted by the thrombosuction device, as well as the indirect consequences of releasing inflammatory molecules during the removal of the occluded thrombus. Furthermore, the extent of concentric vessel wall enhancement is potentially associated with the frequency of device usage, the specific type of device employed, and a heightened risk of hemorrhagic transformation following an infarct.

Compared to conventional angiographic techniques like CT angiography (CTA) or magnetic resonance angiography (MRA), VW-MRI provides a comprehensive evaluation of the vessel wall itself, beyond mere luminal changes. This direct assessment of the vessel wall pathology enables a more accurate diagnosis and facilitates the development of targeted treatment strategies.

## Figures and Tables

**Figure 1:**
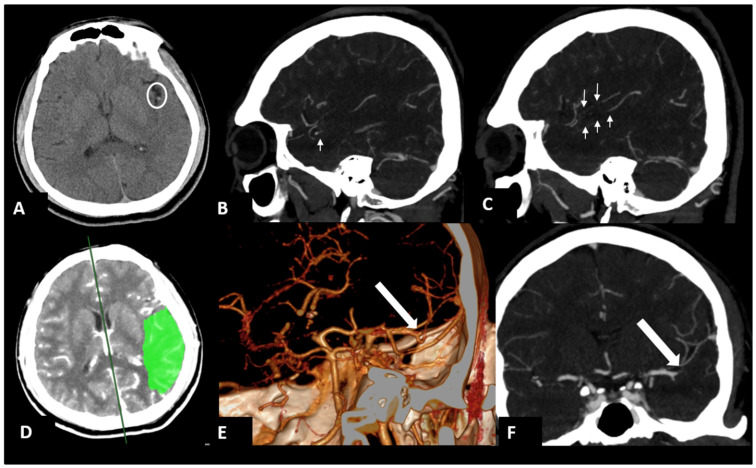
Brain computed tomography (CT) angiography in the ER. The images showed a high-density vessel sign at the left middle cerebral artery (MCA) on axial non-contrast CT (circle in (**A**)) and filling defects in left M2–M3 branches of MCA (arrows) on sagittal maximum intensity projection (MIP) images (**B**,**C**) and volume rendering (**E**) and coronal MIP images (**F**). CT perfusion revealed a large penumbra (green) in the left temporoparietal lobes (**D**).

**Figure 2:**
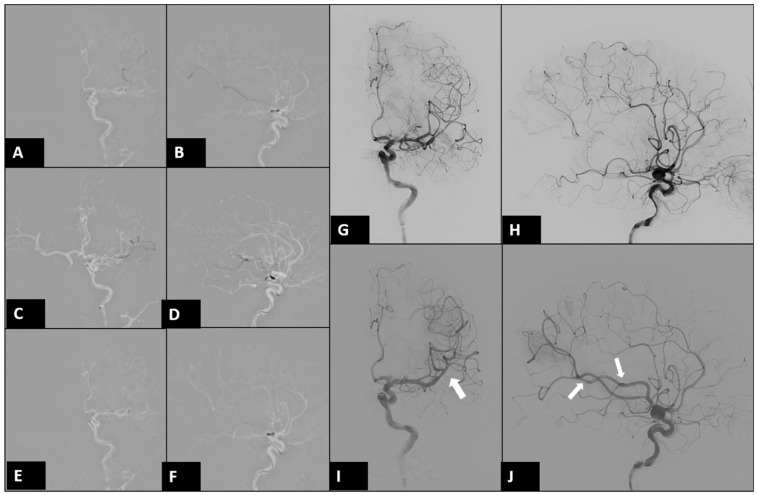
Digital subtraction angiography (DSA) of left internal carotid artery (ICA) at EVT. After it was confirmed that the microcatheter was located beyond the occluded segment in left MCA(**A**–**D**), the stent-assisted EVT with aspiration (SOLUMBRA) method was used (**E**,**F**) [6]. The occluded segment in the left MCA (**G**,**H**) was successfully recanalized after three attempts. (arrows in (**I**,**J**)).

**Figure 3:**
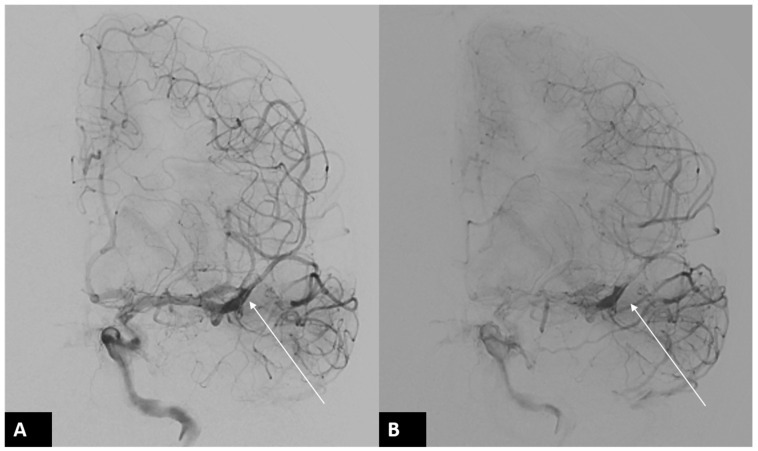
Follow-up DSA of left ICA after EVT. Immediate left internal carotid artery angiogram after successful EVT ((**A**,**B**) arrows) revealed a dissection flap in the proximal M2 segment of the left MCA. Dissection with acute vessel occlusion was diagnosed as the etiology of this patient.

**Figure 4:**
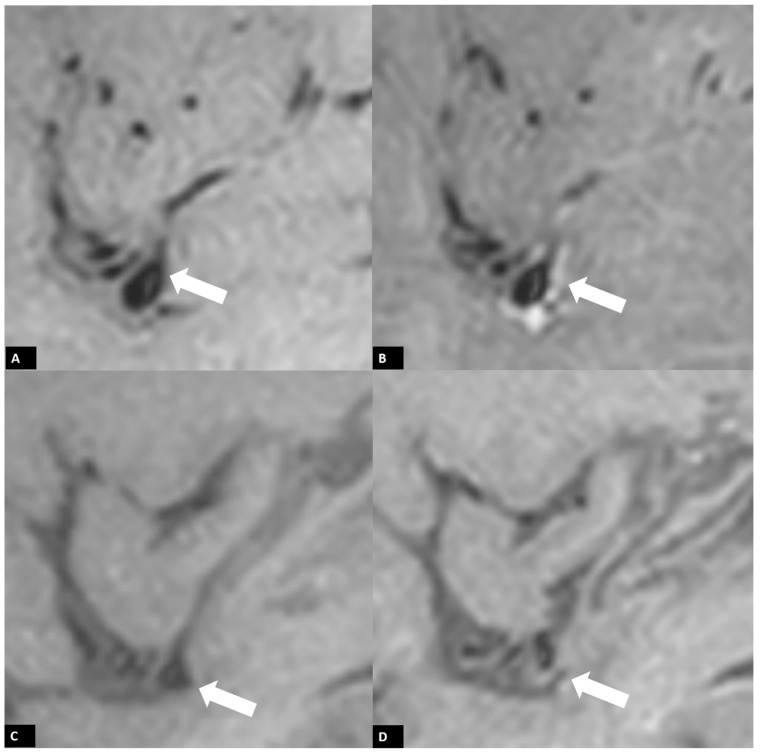
Three-dimensional (3D) black blood T1WI images of vessel wall MRI. A faint high signal on T1WI along the left proximal M2 segment MCA vessel wall due to arterial dissection (arrows) was noted in a follow-up MRI three days after EVT using the pre-contrast sagittal T1WI (**A**) with eccentric enhancement (**B**). Follow-up MRI three months after EVT revealed normalization of the vessel wall enhancement (**D**) and almost normalization of the faint high signal on T1WI (**C**) in the left proximal M2 segment MCA, indicating that the dissection healed with time.

**Figure 5:**
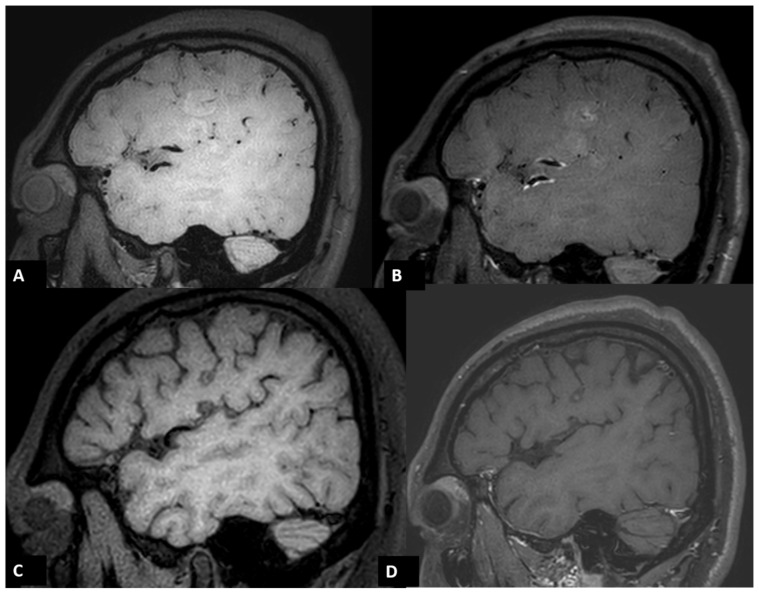
Post-treatment vessel wall enhancement in pre- and post-Gd 3D black blood T1WI images of vessel wall MRI. Concentric vessel wall enhancement of distal left MCA vessel wall (distal to the stent retriever location) (**B**) without abnormal signal on pre-contrast T1WI (**A**) due to post-EVT induced inflammatory process was seen via MRI three days after EVT. The contrast enhancement returned to normal in pre- (**C**) and post-Gd 3D black blood T1WI images (**D**) performed at the three-month follow-up.

## Data Availability

The original contributions presented in the study are included in the article, further inquiries can be directed to the corresponding authors.
